# Gaseous Oxidized Mercury Dry Deposition Measurements in the Southwestern USA: A Comparison between Texas, Eastern Oklahoma, and the Four Corners Area

**DOI:** 10.1155/2014/580723

**Published:** 2014-04-06

**Authors:** Mark E. Sather, Shaibal Mukerjee, Kara L. Allen, Luther Smith, Johnson Mathew, Clarence Jackson, Ryan Callison, Larry Scrapper, April Hathcoat, Jacque Adam, Danielle Keese, Philip Ketcher, Robert Brunette, Jason Karlstrom, Gerard Van der Jagt

**Affiliations:** ^1^Air Quality Analysis Section, US Environmental Protection Agency (EPA) Region 6, 1445 Ross Avenue, Dallas, TX 75202, USA; ^2^National Exposure Research Laboratory, US EPA (E205-03), Research Triangle Park, NC 27711, USA; ^3^Alion Science and Technology, Inc., P.O. Box 12313, Research Triangle Park, NC 27709, USA; ^4^Houston Laboratory, US EPA Region 6, 10625 Fallstone Road, Houston, TX 77099, USA; ^5^Cherokee Nation Environmental Programs, 208 E. Allen Road, Tahlequah, OK 74464, USA; ^6^Eurofins Frontier Global Sciences, 11720 North Creek Parkway North, Suite 400, Bothell, WA 98011, USA

## Abstract

Gaseous oxidized mercury (GOM) dry deposition measurements using aerodynamic surrogate surface passive samplers were collected in central and eastern Texas and eastern Oklahoma, from September 2011 to September 2012. The purpose of this study was to provide an initial characterization of the magnitude and spatial extent of ambient GOM dry deposition in central and eastern Texas for a 12-month period which contained statistically average annual results for precipitation totals, temperature, and wind speed. The research objective was to investigate GOM dry deposition in areas of Texas impacted by emissions from coal-fired utility boilers and compare it with GOM dry deposition measurements previously observed in eastern Oklahoma and the Four Corners area. Annual GOM dry deposition rate estimates were relatively low in Texas, ranging from 0.1 to 0.3 ng/m^2^h at the four Texas monitoring sites, similar to the 0.2 ng/m^2^h annual GOM dry deposition rate estimate recorded at the eastern Oklahoma monitoring site. The Texas and eastern Oklahoma annual GOM dry deposition rate estimates were at least four times lower than the highest annual GOM dry deposition rate estimate previously measured in the more arid bordering western states of New Mexico and Colorado in the Four Corners area.

## 1. Introduction

Atmospheric mercury emissions deposit to the earth through both wet and dry processes, and wet mercury deposition measurements have been taken routinely for over a decade in North America [1–4]. The contribution of atmospheric dry mercury deposition is not as well understood and direct measurements have been mostly limited to short duration research intensives [5–11], and a majority of these recent studies have focused on gaseous oxidized mercury (GOM) dry deposition measurements. The environmental fate of mercury emissions is a function of the physical and chemical properties of the emitted species. Gaseous elemental mercury (GEM) is relatively insoluble and inert, with an atmospheric lifetime of 0.5–2 years leading to global transport [12]. GOM and particulate mercury have much higher deposition velocities leading to local and regional deposition scales [13]. Thus, in many areas a significant portion of total dry deposition of mercury may consist of GOM [14], and GOM can readily deposit to water, soils, and vegetation and is more water soluble than the more abundant GEM constituent [13]. In the arid Four Corners region, GOM dry deposition alone can exceed the estimated total mercury wet deposition [11].

GOM is composed of multiple oxidized mercury compounds such as HgCl_2_ and HgBr_2_ [15], has a short atmospheric life time, and is emitted from local/regional emission sources such as coal-fired power plants and boilers. GOM is also derived from oxidation reactions of gaseous elemental mercury, especially at elevated temperatures during warmer seasons such as spring and summer [14]. Measurements of GOM dry deposition using surrogate surface samplers have been previously evaluated [9, 16, 17] and employed to better understand the spatial distribution of ambient mercury dry deposition [7, 11]. This study gathered one-year GOM dry deposition measurements which were used to assess spatial variability between central and eastern Texas, eastern Oklahoma, and the Four Corners area.

**Figure 1:**
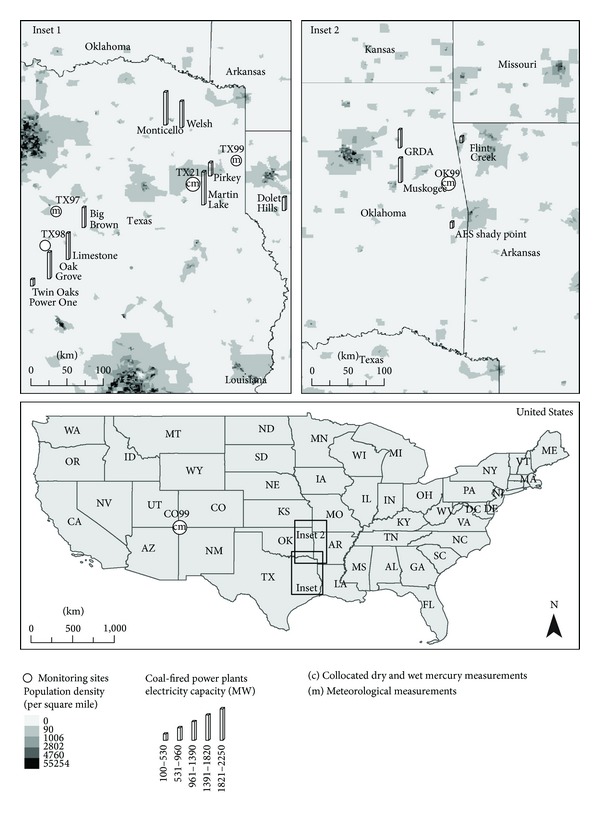
Monitoring sites for the September 27, 2011, to September 25, 2012, Texas/Eastern Oklahoma GOM Dry Deposition Monitoring Study and locations of coal-fired power plants (bottom of bars) within 100 km of the mercury deposition monitoring sites with coal-generated electricity capacity greater than or equal to 100 megawatts (MW). The Mesa Verde National Park site in the Four Corners area (CO99) is included for study comparison purposes.

This paper provides the first GOM dry deposition measurements for the state of Texas in the south central US and compares the measurements derived with previous GOM dry deposition measurements taken in two other south central US areas: (1) the more arid Four Corners area and (2) the similarly humid northern border state of Oklahoma. All these areas are subject to significant coal-fired power plant pollutant emissions. Coal-fired power plants are the greatest anthropogenic mercury emission source in the US and contribute approximately 50% of all stationary source mercury emissions to the atmosphere [18]. Mercury emitted from coal-fired power plants is predominately GEM and GOM, with a smaller contribution from particle bound mercury. Some of the GEM can be oxidized to GOM downwind of the plants, especially in warmer seasons [14]. The speciation/fractionation of the mercury emissions from any power plant is generally dependent on the composition (e.g., halide, sulfur, and ash content) of the coal being burned, the configuration of the boiler, and the installed pollution control equipment. Since central and eastern Texas is similar to eastern Oklahoma in elevation above sea level and annual total precipitation amounts, it was hypothesized that dry mercury deposition estimates for the Texas monitoring sites would be similar to eastern Oklahoma's previously measured dry mercury deposition estimates. The Texas coal-fired power plants in the study domain (Figure 1) used a blend of local lignite and Powder River Basin subbituminous coals, while primarily subbituminous coal was burned in the Four Corners area coal-fired power plants.

The central/east Texas study was a priority project in EPA Region 6 as a response to citizen concerns about a lack of ambient mercury monitoring in areas downwind of coal-fired utility emissions in central/east Texas. The purpose of this study was to characterize GOM dry deposition measurements in the area in terms of both magnitude and influential factors using cost-effective passive monitoring devices.

## 2. Materials and Methods

### 2.1. Study Sites

Dry deposition of GOM was monitored from four sites in central and eastern Texas and one site in eastern Oklahoma (Figure 1). The sites were identified by their names and National Acid Deposition Program (NADP) two letter/two number codes. Site locations were chosen to represent both rural and urban areas downwind of coal-fired power plants in the local and regional areas. Specifically, the sites were located in rural areas (Karnack—TX99—32.669004°N; −94.167449°W, Fort Parker State Park—TX98—31.610783°N; −96.54997°W, and Stilwell, Oklahoma—OK99—35.7514°N; −94.6717°W) and near small-to-medium-sized cities (Longview—TX21—32.37871°N; −94.711834°W, and Corsicana—TX97—32.031944°N; −96.399167°W). The Stilwell site (OK99) contained both the surrogate surface GOM dry deposition passive monitors and a semicontinuous Tekran Instruments Corporation (Toronto, ON) speciation system which provided two-hour integrated measurements of ambient GOM concentrations. The Stilwell site (OK99) also operated for two years during the Four Corners/Eastern Oklahoma GOM dry deposition study [11], so a third year of GOM dry deposition measurements was collected there during this study.

### 2.2. Field Instrumentation for Data Acquisition

Cost efficient and easy to use aerodynamic surrogate surface passive sampling was employed to measure GOM dry deposition during contiguous two-week integrated time periods from September 27, 2011, to September 25, 2012. The use of surrogate surface passive sampling for GOM dry deposition measurements, including deployment of aerodynamic surrogate surface passive samplers, has been discussed in earlier studies [6, 8–11, 19, 20].

The surrogate surface passive sampling conducted in this study employed the Eurofins Frontier Global Sciences (Bothell, WA) Frontier Atmospheric Dry Deposition (FADD) device which uses a negatively charged polysulfone impregnated cation exchange filter membrane (Pall Corporation, ICE 450; 0.45 micron pore size, 140 micron thickness on a nonwoven polymer backing). The FADD device was developed earlier at the University of Nevada [9, 20] and has been tested and shown to selectively and efficiently capture GOM [9]. Each filter membrane was placed into a polyurethane aerodynamic filter holder and mounted approximately 3 m above ground level inverted to avoid contamination from wet deposition.

In addition to surrogate surface passive sampling, a semicontinuous Tekran Speciation system was operated at the Stilwell site (OK99) in eastern Oklahoma by the Cherokee Nation as a part of the NADP's Atmospheric Mercury Network (AMNet) program [2], enabling a collocated correlation analysis with the GOM dry deposition surrogate surface passive monitors. Details of the Tekran Speciation System, including configuration, operation, maintenance, and measurement uncertainties, are presented elsewhere [11, 19, 21, 22].

Hourly meteorological data were collected at four of the five sites (Figure 1). The meteorological data were collected by the Texas Commission on Environmental Quality for sites TX21, TX97, and TX99 and by the Cherokee Nation for the Stilwell site (OK99). Weekly integrated total mercury wet deposition measurements from the NADP's Mercury Deposition Network (MDN) [23] were collected at two of the five sites (Figure 1), enabling conservative estimates of total mercury deposition at those two sites. Total mercury deposition is defined in this paper as the total of GOM dry deposition estimates plus total mercury wet deposition estimates only, not including dry measurements of particle-bound or elemental gaseous mercury.

### 2.3. Laboratory Procedures

#### 2.3.1. Sample Preparation and Handling

All samples were prepared and sent to the field in identical fashion to the earlier two-year Four Corners/Eastern Oklahoma study [11]. Field blanks travelled to each site and at each of the five sites duplicate field sampling was planned for every other sampling period to evaluate sampling precision, and duplicate field blank sampling was conducted at the initial sampling period and then once every four sampling periods thereafter. Additional precision sampling became possible, and duplicate field samples at each site were deployed in 18 of the 26 two-week sampling periods, resulting in 69% of the study containing precision sampling. Field blank data were tracked throughout the study and were subtracted from the field sample data at each site [11]. For each two-week sampling period with duplicate field sampling, the final GOM dry deposition estimate was calculated as the arithmetic mean of the two duplicate field samples.

#### 2.3.2. Chemical Analyses

All samples were chemically analyzed at Eurofin Frontier Global Sciences in the identical fashion to the earlier two-year Four Corners/Eastern Oklahoma study [11]. Frontier Global Sciences employed cold vapor atomic fluorescence spectroscopy (CVAFS) for chemical analysis following Frontier Standard Operating Procedure (SOP) FGS-069, based on the principles of US EPA Method 1631 revision E [24] and additional experimental quality assurance procedures for mercury analysis [25, 26].

### 2.4. Statistical Analyses

The following statistical methods were applied to the data resulting from this project: relative percent difference (RPD), 95% confidence interval, and stepwise linear regression. The detection limit for the aerodynamic surrogate surface passive GOM dry deposition sampling using the FADD filter membranes for this one-year study was calculated as three times the standard deviation of the field blanks. The precision for the one-year study was reviewed by calculating relative percent difference (RPD) values of all FADD filter membrane field sample duplicates using

(1) RPD=[absolute  difference  of  field  sample  duplicatesaverage  of  field  sample  duplicates] ∗100%.

Site comparisons were conducted by calculating 95% confidence intervals about the estimated means. Given the overall length of the study, the confidence intervals were based on the application of the central limit theorem.

Dry deposition is a product of deposition velocity and concentration as presented in (2) below [27]:

(2) Dry  deposition  of  GOM  =GOM  concentration   ∗GOM  dry  deposition  velocity  (Vd),

where *V*_*d*_ is calculated via the big-leaf dry deposition model as described in Zhang et al. [28]: *V*_*d*_ = 1/(*R*_*a*_ + *R*_*b*_ + *R*_*c*_) where *R*_*a*_ is aerodynamic resistance, *R*_*b*_ is quasilaminar resistance, and *R*_*c*_ is canopy resistance.

*R*_*a*_ and *R*_*b*_ are influenced by atmospheric turbulence such as wind speed and *R*_*c*_ is affected by meteorological and surface conditions such as temperature and precipitation [9]. Both temperature and wind speed are commonly measured meteorological variables and were measured as part of this study. But other potentially influential variables (e.g., surface wetness and humidity) are often not available (and were not here).

To examine the capability of the meteorological variables measured in this study to predict mercury deposition levels over the two-week time frame of the passive sampling period, stepwise linear regression was performed on a site-by-site basis using the REG procedure in SAS (Cary, NC) Version 9.3 (preparation of meteorological data for use in the regressions is described below.) The regressions were done for both GOM dry deposition and total mercury wet deposition data. For each site, a wind sector was designated as a power plant wind sector if one or more power plants (within 100 km) were located in that sector. Plotting and correlation calculations were used to screen the large number of potential predictor variables for candidates to use in the regressions.

For each site, the initial set of potential predictors included the fraction of hours from each power plant wind sector and the fraction of precipitation for each of these. In addition, any other wind sectors for which fraction of time or fraction of precipitation, average wind speed, average temperature, or total precipitation for which the preliminary plotting or correlation calculations suggested might be influential were also included as initial variables for the stepwise regressions. Correlations and plots for the deposition variables were very similar between the average overall temperature and the average day and night temperatures; therefore, when temperature was employed, the overall average temperature was used (with the one exception being the Valles Caldera National Preserve (NM97) site in New Mexico). A variation on this approach was also employed. After these regressions were done, they were repeated, but with the sum over all power plant sectors of the wind direction fractions and precipitation fractions substituting for the set of individual sectors. These repeat regressions were only conducted for sites with more than one power plant sector. The SAS default of a probability of 0.15 to enter the model was used. Residual analyses and checks for collinearity, autocorrelation, and homoscedasticity were done for each regression.

The potential use of power plant sectors relied on the assumption that the wind direction recorded at the monitoring site corresponded to some extent with the wind direction at the power plants. Evaluation of the validity of this assumption and the use of other wind direction sectors in the regressions are presented in the supplemental information; see Supplementary Materials available online at http://dx.doi.org/10.1155/2014/580723.

### 2.5. Meteorological Data Preparation and Analyses

GOM dry deposition and total wet deposition mercury data from the Texas/Oklahoma sites and from the Four Corners area sites discussed in Sather et al. [11], along with the accompanying meteorological data, were subjected to detailed statistical analyses. Hourly wind speed, wind direction, ambient temperature, and precipitation amounts were collected at each monitoring site, with a few exceptions. No meteorological data were collected at the Farmington Airport (NM99) or Fort Parker State Park (TX98) sites (see [11] for a complete map of Four Corners area site locations). The Karnack site (TX99) did not collect precipitation data, but hourly precipitation amounts were obtained from a nearby (approximately 0.10 km away) US Fish and Wildlife Service site at Caddo Lake. The Navajo Lake site (NM98) did not collect hourly precipitation, but weekly precipitation amounts from the NADP were available. Only precipitation amounts were collected at the Molas Pass site (CO96), and this was also on a weekly basis from the NADP. There were no precipitation data available from the Corsicana (TX97) or Substation (NM95) sites. Each site collected GOM dry deposition measurements, and six of the sites also collected total wet deposition mercury measurements. The sites which did not collect total wet deposition mercury measurements were Substation (NM95), Farmington Airport (NM99), Karnack (TX99), Corsicana (TX97), and Fort Parker State Park (TX98).

In preparation for the regression analyses, the meteorological data were summarized over the dry deposition sampling periods with matching done to the nearest beginning and ending hours. For the two sites with only weekly precipitation data, the matching was to the nearest day for this measurement; however, study protocol called for the start and end of the dry and wet deposition sampling periods to agree as closely as possible. The meteorological data were summarized over the dry deposition sampling periods as follows: (1) total precipitation and average wind speed were calculated; (2) temperatures were summarized as the average daytime (fixed as 7 am to 6 pm at all sites), average nighttime (i.e., nondaytime), and average overall (i.e., no day or night distinction) temperatures; (3) for wind direction data, the compass was divided into eight sectors: NNE, ENE, ESE, SSE, SSW, WSW, WNW, and NNW. The hourly wind direction reported in degrees was assigned to one of these eight sectors, and the fraction of hours assigned to each sector was calculated for each dry deposition sampling period. In addition, the fraction of precipitation corresponding to each wind sector was determined.

No significant departures from model assumptions were encountered for each regression when evaluated for collinearity, autocorrelation, and homoscedasticity. In a few instances, heteroscedasticity was suggested by the rejection of homoscedasticity at the 10% significance level; in such cases, the *P* values for the entry of predictors into the model are based on asymptotically consistent results adjusting for heteroscedasticity. Though some moderate (or large, for one period at one site) residuals were occasionally present, no valid data were excluded from any regression.

In addition to analyzing the available local ground level meteorological data, back trajectory analyses were conducted to examine mesoscale meteorological effects. Specifically, back trajectory analyses were conducted for the three highest GOM dry deposition two-week sampling periods at the highest Texas GOM dry deposition site at Corsicana (TX97). The National Oceanic and Atmospheric Administration (NOAA) HYSPLIT model [29] was used to create seven 48-hour back trajectories encompassing each two-week sampling period. Each back trajectory used the Eta Data Assimilation System (EDAS) meteorological data resident in the HYSPLIT model and was conducted at a starting height of 500 meters above ground level.

## 3. Results and Discussion

### 3.1. Detection Limit, Precision, and Comparison of Surrogate Surface Passive Sampling

Based upon a 0.0102 m^2^ exposure area of the surrogate surfaces, the average five-site GOM dry deposition detection limit was 0.13 ng/m^2^h, similar to the 0.12 ng/m^2^h detection limit reported earlier for the Four Corners/Eastern Oklahoma study [11]. All field samples collected by the aerodynamic surrogate surface passive samplers were at or above the detection limit except for one sample at Fort Parker State Park (TX98) and two samples at Karnack (TX99). The average FADD field blank was 0.21 ng/filter membrane, compared to an average FADD laboratory blank of 0.18 ng/filter membrane. The average ambient sample GOM loadings for the one-year study were 0.7 ng/filter membrane at the Fort Parker State Park site (TX98) and 1.29 ng/filter membrane at the Corsicana site (TX97), representing the lowest and highest GOM dry deposition sites, respectively. All final two-week GOM dry deposition estimates were derived by subtracting site specific field blank estimate data from ambient sample data.

For all of the field duplicate samples (*N* = 88), 68% had relative percent differences (RPDs) ≤20%, with RPD increasing for lower GOM dry deposition estimates. The median RPD for the study was 13.5% which compared favorably with the median RPD of 10% for the earlier two-year Four Corners/Eastern Oklahoma study [11]. A higher median RPD was expected for this study because of the lower GOM dry deposition estimates recorded by the samplers compared to the Four Corners area sites. The mean RPD for this study was 19.6% with a standard deviation of 20.3% and a minimum/maximum RPD of 0% and 106%, respectively.

The aerodynamic surrogate surface passive GOM dry deposition results were compared to collocated Tekran GOM ambient concentrations at the Stilwell (OK99) site. As indicated in (2), the dry deposition of GOM should be directly proportional to ambient concentration and should be a reasonable quality assurance comparison. This correlation analysis was also done during the earlier two-year Four Corners/Eastern Oklahoma study [11]. For the current study, the GOM dry deposition rate estimate data was correlated with the GOM ambient concentration data at the same correlation coefficient of *r* = 0.6. This is similar to correlations at other low GOM ambient concentration sites reported previously [6, 9, 10].

### 3.2. GOM Dry Deposition Measurements

#### 3.2.1. Temporal and Spatial Analysis

**Figure 2:**
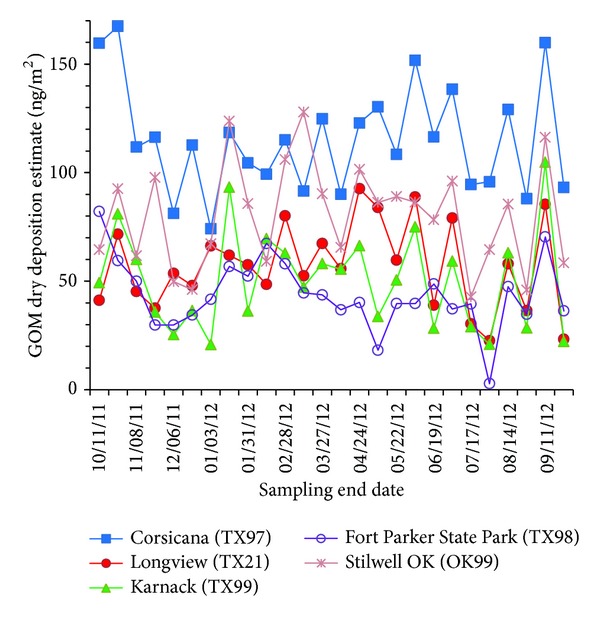
GOM dry deposition data time series for the Texas and Stilwell, Oklahoma sites; September 27, 2011, to September 25, 2012.

The GOM dry deposition estimates data time series (Figure 2) for the four Texas sites and the Stilwell, Oklahoma site (OK99) showed no significant seasonal differences. Comparing the data across all sites showed low coefficients of determination between all of the sites (Table 1), which differed from the medium to high coefficients of determination seen at the Four Corners sites in New Mexico and Colorado [11]. The low coefficients of determination between the Texas sites and with Stilwell (OK99) reflect the lower GOM dry deposition estimates recorded and perhaps also suggest spatial differences and source sensitivity as well, relative to the Four Corners area.

Mean GOM dry deposition estimates were calculated for each of the five sites for the study year. Using 95% confidence intervals, the one-year mean of all 2-week integrated GOM dry deposition estimates for the Corsicana site (TX97) at 115 ng/m^2^ was significantly higher than the other three Texas sites' annual mean GOM dry deposition estimates (ranging from 44 to 57 ng/m^2^) but was not significantly different from the Stilwell, Oklahoma site (OK99) annual mean GOM dry deposition estimate of 80 ng/m^2^. The other three Texas sites (TX21, TX98, and TX99) GOM dry deposition estimate annual means were not significantly different from the Stilwell, Oklahoma site (OK99) annual mean GOM dry deposition estimate. The precipitation totals and ambient temperature and resultant wind speed arithmetic means for the Longview site (TX21) during the study were compared to longer term (i.e., 7 years from 2006 to 2012) annual averages to acquire context for the one-year study results. For all three parameters (precipitation totals, mean ambient temperature, and mean resultant wind speed), the one-year study statistics for the Longview site (TX21) were within the 95% confidence intervals for the 7-year averages. In summary, the Corsicana site in central Texas recorded GOM dry deposition estimates about two times higher (and statistically significant at the 95% confidence interval) than the other central Texas site and both east Texas sites. This is similar to the Four Corners area where one site at Mesa Verde National Park (CO99) consistently recorded the highest GOM dry deposition measurements relative to the other five sites in the area, though, on the other hand, the high elevation Molas Pass site (CO96) had consistently lower levels relative to the other five sites in the area [11].

Annual GOM dry deposition estimates, mercury wet deposition [23], and conservative total mercury deposition estimates (if available) for the Texas sites and the eastern Oklahoma site are presented in Table 2. Also listed in Table 2 are elevation, precipitation, and coal-fired power plant electricity capacity data. Note the comparable elevation and precipitation data reported for the Texas monitoring sites and the eastern Oklahoma site. In addition, there is notable modified coal-fired power plant electricity capacity within 100 km of each monitoring site that is emitting mercury in both Texas and eastern Oklahoma: 3405 MW-Hg at the eastern Oklahoma Stilwell site and ranging from 1964 MW-Hg to 3322 MW-Hg at the Texas monitoring sites. Primary emissions from the coal-fired power plants are GEM and GOM, with some of the GEM possibly oxidized to GOM downwind of the plants. The modified coal-fired power plant electricity capacity that is outputting mercury emissions was calculated by taking the referenced coal-fired power plant electricity capacity [30] for each plant within 100 km of each monitoring site and taking into account any mercury emission controls put into place between 2009 and 2011 before our sampling studies. In the Four Corners area the San Juan Power Plant had installed approximately 80% mercury control with activated carbon injection in 2009 before the August, 2009–August, 2011 monitoring study began. Likewise in Texas, the Oak Grove Power Plant installed approximately 90% mercury control with activated carbon injection in 2009, and three additional power plants (Big Brown, Martin Lake, and Monticello) installed approximately 90% mercury control with activated carbon injection in 2011, all before this September 27, 2011–September 25, 2012 monitoring study. Despite the significant power plant mercury emissions, recorded GOM dry deposition estimates were uniformly much lower at all of the Texas monitoring sites compared to the Four Corners monitoring sites. This was not surprising since the Texas sites all reside in more humid areas that receive significantly more amounts of rainfall than the sites in the Four Corners area. Thus, wet deposition of mercury, instead of dry deposition of GOM, dominates at the Texas sites.

#### 3.2.2. Comparison with Other Extended Length US Studies

Annual data summaries from previous extended length GOM dry deposition studies conducted in the US in the Four Corners area (New Mexico and Colorado), Nevada, Georgia, Florida, and Maryland [6, 9–11] are presented in Table 2. The four Texas sites' GOM dry deposition estimates and hourly rate estimates were low and similar to the GOM dry deposition estimates and hourly rate estimates recorded at the eastern Oklahoma Stilwell site (OK99) and other sites in the States of Georgia and Florida. The GOM dry deposition hourly rate estimates for the Texas sites were four to twelve times lower than the GOM dry deposition hourly rate estimate recorded at the more arid Mesa Verde National Park (CO99) and Reno, Nevada sites. Wet deposition dominated the Longview site's (TX21) total mercury deposition estimate, with the GOM dry deposition estimate for the Longview site (TX21) contributing a low percentage of 11% to the total mercury deposition estimate for the one-year study. The wet deposition domination seen at the Texas Longview site (TX21) and eastern Oklahoma Stilwell site (OK99) has also been reported at other sites in the eastern US [9, 10, 31].

#### 3.2.3. Statistical Analyses of Deposition and Meteorological Data from Texas, Eastern Oklahoma, and Four Corners Area Sites

To assess the influence of meteorology and local/regional mercury emission sources such as coal-fired utility boilers on the recorded GOM dry deposition and mercury wet deposition measurements, stepwise linear regression modeling was employed for sites in Texas, Eastern Oklahoma, and in the Four Corners area. The results from the regression modeling analyses are presented in Table 3.

In the Four Corners area, where GOM dry deposition composes almost half of the total mercury deposition (i.e., 40–51% based on a two-year average data set), the meteorological data predictors in the model accounted for 62–72% of the variability of the GOM dry deposition recorded measurements. As suggested by (2), temperature and wind speed were the most important model predictors in the Four Corners area. At two sites, winds from power plant sectors or adjacent sectors were also useful predictors. As would be expected, precipitation amount was the most important predictor for wet deposition in the Four Corners region. However, wind sectors were also important predictors at Mesa Verde (CO99), Navajo Lake (NM98), and Valles Caldera (NM97) (where nighttime temperature was also found to be predictive).

As previously surmised [11], the sources for the GOM dry deposition and wet deposition data are suggested to be from multiple areas, including local/regional coal-fired power plants and boilers and natural/global sources such as possibly subsiding air from the free troposphere. Elevated temperatures could lead to more oxidation of gaseous elemental mercury to GOM, and increased wind speeds reflect more atmospheric turbulence which should increase GOM deposition rates.

GOM dry deposition was very poorly predicted at each of the Texas and Oklahoma sites. Surprisingly, in contrast to the Four Corners results, neither temperature nor wind speed were of much, if any, importance in this regard. While the Texas sites were sampled for only one year, whereas two years of data were available from the Four Corners sites, this would not seem to be a viable explanation for this outcome because three years of data were collected at Stilwell, Oklahoma. Another possibility might be that linear modeling did not detect the nonlinear relationship of dry deposition with temperature and wind speed; however, the residual analyses did not suggest a lack of fit from the linear model for these two week data. Perhaps this result is attributable to a variable that was omitted from the regressions. For example, the region from central Texas to eastern Oklahoma is more humid than the Four Corners area, and humidity was not included in the models here. Another distinction between the two regions is that GOM dry deposition was quite low relative to total mercury wet deposition at the Longview, Texas (TX21) and Stilwell, Oklahoma (OK99) sites for which both wet and dry deposition were monitored. But, as Table 3 reports, total precipitation was not an important predictor of GOM dry deposition at any site in Texas or Oklahoma (precipitation fraction from a power plant sector did enter the predictive equation at OK99, but the *r* ^2^ value was only 6%). In any case, the different results obtained with respect to the effect or lack of effect of temperature and wind speed on GOM dry deposition suggests the need for further investigation of this, including more ambient monitoring, in less arid (more humid) regions.

**Figure 3:**
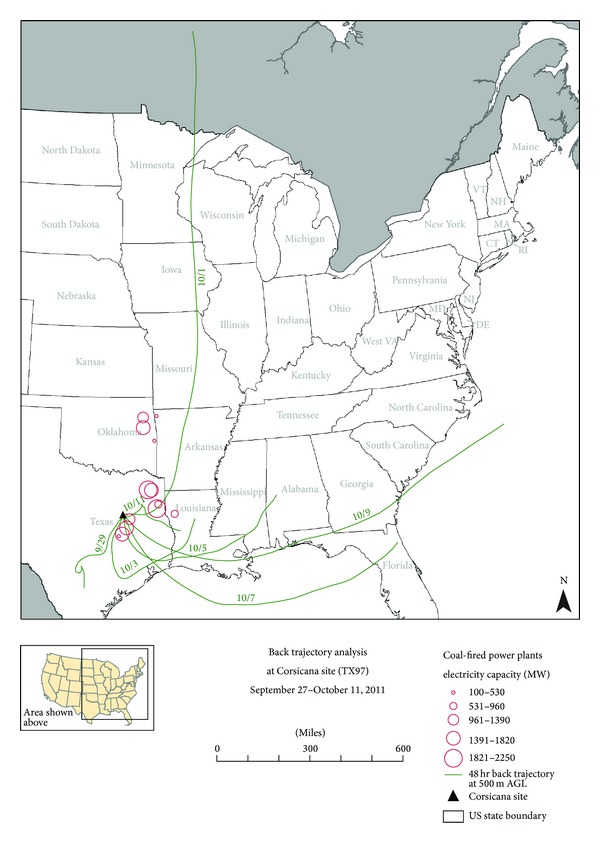
Back trajectory analysis for the Corsicana site (TX97) for September 27 to October 11, 2011. Seven contiguous 48-hour back trajectories ending at 1100 LST on October 11, 2011. End date of each 48-hour back trajectory plotted for each trajectory trace (e.g., 9/29 represents 48-hour back trajectory for 9/27–9/29). Coal-fired power plant locations are located at center of open circles.

**Figure 4:**
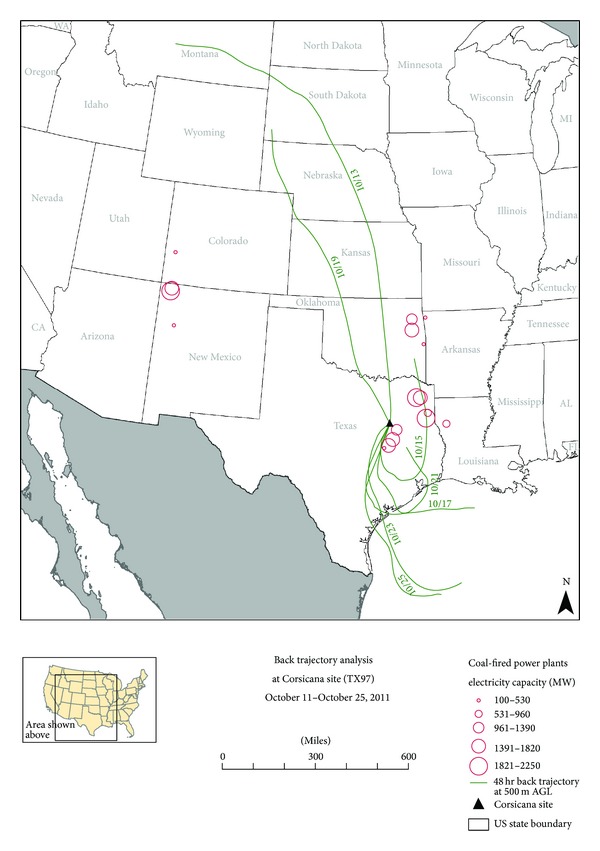
Back trajectory analysis for the Corsicana site (TX97) for October 11 to October 25, 2011. Seven contiguous 48-hour back trajectories ending at 1000 LST on October 25, 2011. End date of each 48-hour back trajectory plotted for each trajectory trace (e.g., 10/13 represents 48-hour back trajectory for 10/11–10/13). Coal-fired power plant locations are located at center of open circles.

**Figure 5:**
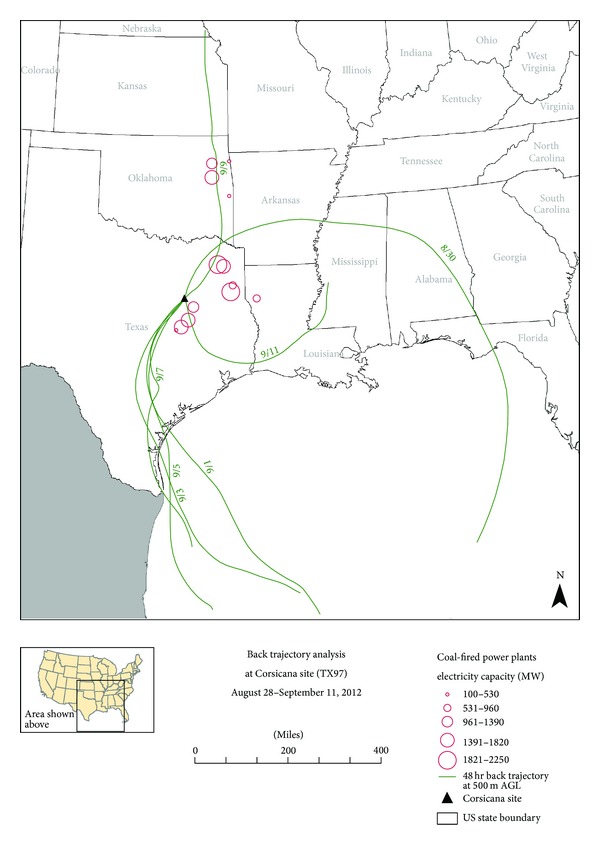
Back trajectory analysis for the Corsicana site (TX97) for August 28 to September 11, 2012. Seven contiguous 48-hour back trajectories ending at 1000 LST on September 11, 2012. End date of each 48-hour back trajectory plotted for each trajectory trace (e.g., 8/30 represents 48-hour back trajectory for 8/28–8/30). Coal-fired power plant locations are located at center of open circles.

The back trajectory analyses for the top three GOM dry deposition days at the Corsicana site (TX97) produced both similar and different results than those obtained for the top three GOM dry deposition days at the Mesa Verde National Park site (CO99). In summary for the Mesa Verde National Park site (CO99) back trajectories, some, but not all, of the air masses passed proximal to local/regional coal fired power plants before arriving at the site, similar to the back trajectories produced for the Corsicana (TX97) site (Figures 3, 4, and 5). This suggests multiple mercury emission sources, including local/regional coal-fired power plants, may be impacting the mercury deposition monitoring sites. The difference in trajectory maps between the Mesa Verde National Park site (CO99) and the Corsicana, Texas site (TX97) was that for Mesa Verde National Park (CO99), all back trajectories passed over the Four Corners area or other areas in the western US, not other areas in the central or eastern US. In contrast to this, the back trajectories analyzed for the Corsicana, Texas site (TX97) primarily passed over areas in Texas, the more humid southeastern Gulf Coast states of the US, and the Gulf of Mexico itself, with some input from the north central states above Texas. Having more humid air masses impacting the Corsicana site (TX97) could also help explain the lower GOM dry deposition data recorded at the Corsicana site (TX97) during the course of the study.

The Texas Longview site (TX21) and eastern Oklahoma site at Stilwell (OK99) were both dominated by wet mercury deposition (Table 2). For wet mercury deposition, the meteorological data predictors in the model accounted for 59% (TX21) and 62% (OK99) of the variability. Not surprisingly the precipitation variable was the strongest model predictor for wet mercury deposition, but winds from the power plant sectors were also significant, suggesting some impact from those sources.

## 4. Conclusions

This study has provided the first long term gaseous oxidized mercury (GOM) dry deposition monitoring data in central and eastern Texas and provided a third consecutive year of GOM dry deposition monitoring data at a site in eastern Oklahoma. The Texas sites were hypothesized to have low portions of their total atmospheric mercury deposition occur via dry processes, similar to the eastern Oklahoma Stilwell site (OK99). Indeed, mercury dry deposition (conservatively represented by the GOM dry deposition measurements) contributed a low percentage of 11% to the September 27, 2011–September 25, 2012 one-year total mercury deposition estimate at the Longview site (TX21) in east Texas. The Stilwell (OK99) site in eastern Oklahoma was also dominated by wet mercury deposition for a third consecutive year, with the GOM dry deposition estimate contributing only 17% to the one-year total mercury deposition estimate at that site. Since only GOM dry deposition is estimated in this paper, the total mercury deposition estimates discussed are conservative (i.e., underestimates) because they do not include complete dry deposition inputs from particle bound mercury and GEM. All four of the Texas sites and the eastern Oklahoma site at Stilwell (OK99) recorded GOM dry deposition hourly rate estimates that were generally uniform across all of the sites and that were four to twelve times lower than the highest Four Corners area site at Mesa Verde National Park (CO99) in southwest Colorado, where GOM dry deposition represented 57% of the annual total mercury deposition estimate at that site for the one-year period of August, 2010–August, 2011. One site in central Texas (Corsicana) recorded GOM dry deposition estimates about two times higher (and statistically significant at the 95% confidence interval) than the other central Texas site and both east Texas sites. In the Four Corners area, one site (Mesa Verde National Park) consistently recorded the highest, and one site (Molas Pass) the lowest, GOM dry deposition measurements.

Linear regression modeling and back trajectory analysis support the premise that multiple mercury sources (local/regional/natural/global) were impacting the GOM dry deposition and total mercury wet deposition measurements. The degree of influence of those sources, though, still has uncertainty, and follow-up GOM dry deposition measurements after the full implementation of the 90% mercury emissions control on power plants and certain boilers should help provide information to address that question. As a scientific implication from analysis of the GOM dry deposition monitoring data in the Four Corners area, eastern Oklahoma, and central/eastern Texas, it is recommended that the follow-up GOM dry deposition measurements occur in the Four Corners area. This is where the highest GOM dry deposition signal was detected versus eastern Oklahoma and the central/eastern portions of Texas which were dominated by wet deposition of mercury.

## Supplementary Material

This supplemental material provides an explanation of the wind direction sectors used in the linear regression data analyses.

## Figures and Tables

**Table 1:** Coefficients of determination (*r* ^2^) for Texas and Stilwell, Oklahoma GOM smooth-edge surrogate surface passive sampling sites. All values significant at *P* < 0.05 except as noted.

Site (across and down)	Corsicana (TX97)	Longview (TX21)	Karnack (TX99)	Fort Parker State Park (TX98)	Stilwell, Oklahoma (OK99)
Corsicana (TX97)	—	0.26	0.43	0.21	0.18
Longview (TX21)	0.26	—	0.38	0.04 (not significant)	0.34
Karnack (TX99)	0.43	0.38	—	0.36	0.37
Fort Parker State Park (TX98)	0.21	0.04 (not significant)	0.36	—	0.06 (not significant)
Stilwell, Oklahoma (OK99)	0.18	0.34	0.37	0.06 (not significant)	—

**Table 2:** Annual GOM dry deposition (dep.) estimates and mercury wet deposition data, elevation, precipitation, and modified coal-fired power plant electricity capacity data for Texas and Eastern Oklahoma sites and other comparison sites, September 27, 2011-September 25, 2012 for Texas and Eastern Oklahoma sites (except as noted); asl: above sea level; na: not available; h: hour; Hg: mercury; total mercury deposition estimates = GOM dry deposition estimates + mercury wet deposition data; comparison of GOM data for 10/06–10/08 sites could be higher by 0.2 ng/m^2^h.

Site	Elevation (asl)	Precipitation (mm)	Surrogate surface dep. rate estimate (ng/m^2^h) ± standard deviation	GOM dry dep. estimate (ng/m^2^)	Mercury wet dep. (ng/m^2^)	GOM dry dep. + mercury wet dep. estimates (ng/m^2^)	GOM dry dep. % of total mercury dep. estimate	Total modified coal-fired power plant electricity capacity (MW) which is outputting Hg emissions within 100 km of sites (Texas and Eastern Oklahoma and Mesa Verde National Park sites only)
Corsicana (TX97)	128 m	na	0.3 ± 0.1	2996	na	na	na	1964 MW-Hg
Longview (TX21)	110 m	1158	0.2 ± 0.1	1486	11902	13388	11	2672 MW-Hg
Karnack (TX99)	85 m	1074	0.2 ± 0.1	1313	na	na	na	3322 MW-Hg
Fort Parker State Park (TX98)	163 m	na	0.1 ± 0.0	1142	na	na	na	2269 MW-Hg
Stilwell (OK99)	304 m	1010	0.2 ± 0.1	2089	9869	11958	17	3405 MW-Hg
Stilwell (OK99)—8/09-8/10; Sather et al. 2013 [11]	304 m	1591	0.1 ± 0.1	1118	13452	14570	8	3405 MW-Hg
Stilwell (OK99)—8/10-8/11; Sather et al. 2013 [11]	304 m	1247	0.3 ± 0.1	2350	13263	15613	15	3405 MW-Hg
Annual comparison sites								
Mesa Verde National Park (CO99)—highest annual GOM dry deposition estimate site in Four Corners area (8/10-8/11; Sather et al. 2013) [11]	2172 m	368	1.2 ± 0.7	10889	8289	19178	57	2409 MW-Hg
Reno, Nevada (10/06–10/08; Lyman et al. 2009) [9]	1340 m	59	1.0 ± 0.8	6800	1500	8300	82	
Yorkville, Georgia (10/06–10/08; Lyman et al. 2009) [9]	394 m	1175	0.2 ± 0.2	1900	10700	12600	15	
Tampa, Florida (7/09-7/10; Peterson et al. 2012) [10]	4 m	1248 mean estimate	0.2 ± 0.1	2949	18217	21166	14	
Pensacola, Florida (10/06–10/08; Lyman et al. 2009) [9]	44 m	1791	0.1 ± 0.1	700	13600	14300	5	
Western Maryland (9/09-9/10; Castro et al. 2012) [6]	869 m	not given	0.4 estimated	2530	7700	10230	25	

**Table 3:** Regression results: coefficients of determination (*r* ^2^) and sample size (*n*) for meteorological data modeling for Texas, Oklahoma, and Four Corners area sites.^a^

Site	Dry *r* ^2^ (*n*)	Model dry predictors	Wet *r* ^2^ (*n*)	Model wet predictors
Corsicana (TX97)	0.12 (24)	TEMP* (+)	na	na
Longview (TX21)	0.14 (26)	WS (+)	0.59 (26)	RAIN*** (+) >> Wind_PP*** (+)
Karnack (TX99)	0.00 (25)	None	na	na
Stilwell, Oklahoma (OK99)	0.06 (78)	R_WNW**_p_ (+)	0.62 (78)	RAIN*** (+) >> W_SSE*_p_ (+)
Substation (NM95)	0.64 (44)	TEMP*** (+) >> W_WSW*** (+) > W_NNE (−)	na	na
Mesa Verde National Park (CO99)	0.62 (44)	WS (+) >> TEMP** (+), W_ENE* (−) > R_SSE*_p_ (−), W_ESE (−), W_SSW (+)	0.43 (46)	RAIN*** (+) >> R_SSE**_p_ (+), W_NNE** (−)
Valles Caldera National Preserve (NM97)	0.72 (42)	WS*** (+) >> DTEMP** (+) > R_WNW** (+), W_WSW* (+)	0.75 (41)	RAIN*** (+) >> NTEMP*** (+) > R_SSE** (+), R_NNW* (+)
Navajo Lake (NM98)	0.65 (47)	W_ESE (−) >>W_ENE*** (−), TEMP*** (+), WS (+)	0.56 (46)	RAIN*** (+) >> W_ENE*** (+), W_NNW* (+)

^a^Corsicana (TX97), Karnack (TX99), and Substation (NM95) sites collected GOM dry deposition data only; thus the wet *r* ^2^ and wet model predictors were not applicable (na). All meteorological variables entered the model at the 0.15 *P* level. Asterisks denote more significant *P* levels as *0.10 *P*-level, **0.05 *P*-level, and ***0.01 *P* level. Model predictors are listed in order of rank based on their contribution to the final model's explanatory power as indicated by their partial *r* ^2^ values; >> and > indicate distinctions between the partial *r* ^2^ of the predictors. The direction of influence on the deposition variable is indicated by a + or − sign. Wind sector predictors are designated as W_XXX or R_XXX to indicate the fraction of time or precipitation, respectively, associated with the XXX sector; a subscript of *p* indicates a power plant sector. The combined power plant sectors are designated as WIND_PP (or RAIN_PP). The other variables are designated as WS for average wind speed; TEMP, DTEMP, or NTEMP for overall, daytime, and nighttime average temperature, respectively; RAIN for total precipitation amount.
