# Supplementary material for: Gaseous Oxidized Mercury Dry Deposition Measurements in the Southwestern USA: A Comparison between Texas, Eastern Oklahoma, and the Four Corners Area
Source: ScientificWorldJournal. 2014 Apr 6;2014:580723. doi: 10.1155/2014/580723 (PMC3997905; doi:10.1155/2014/580723)
Supplement: Supplementary file 1 — This supplemental material provides an explanation of the wind direction sectors used in the linear regression data analyses. [file 580723.f1.pdf]

Supplemental Information. ***Additional information regarding the potential use of power plant and other wind direction sectors.***

While no wind direction data were available from the power plants themselves, the assumption that the wind direction at the monitoring site was to some extent indicative of wind direction from the power plants was approximately evaluated by the following procedure. The Substation (NM95), Mesa Verde National Park (CO99), and the Navajo Lake (NM98) sites all contained only the San Juan and Four Corners power plants within 100 km. Furthermore, the Substation site (NM95) was very close to the San Juan and Four Corners plants (4 and 12 km, respectively), and these two plants were both in a single wind sector for the Mesa Verde National Park (CO99) and Navajo Lake (NM98) sites (SSE and WSW, respectively). The separation distances for the San Juan and Four Corners plants were 43 and 56 km, respectively, for Mesa Verde National Park (CO99), and 70 and 76 km, respectively, for Navajo Lake (NM98). Given these relationships, the Substation site (NM95) was used as a surrogate for the wind direction at these two power plants, and the correlations of wind sector time fractions between the Substation site (NM95) and Mesa Verde (CO99) and Navajo Lake (NM98) sites were calculated. The results were a correlation of 40% for the SSE power plant sector at Mesa Verde (CO99), and 26% and 30% at the adjacent SSW and ESE sectors, respectively. For Navajo Lake (NM98), the correlation with the WSW power plant sector was only 16%, but was 45% for the adjacent WNW sector. These results suggested that other wind sectors beyond those containing power plants be considered for use in the regressions.
